# Sampling-Based Approaches to Improve Estimation of Mortality among Patient Dropouts: Experience from a Large PEPFAR-Funded Program in Western Kenya

**DOI:** 10.1371/journal.pone.0003843

**Published:** 2008-12-02

**Authors:** Constantin T. Yiannoutsos, Ming-Wen An, Constantine E. Frangakis, Beverly S. Musick, Paula Braitstein, Kara Wools-Kaloustian, Daniel Ochieng, Jeffrey N. Martin, Melanie C. Bacon, Vincent Ochieng, Sylvester Kimaiyo

**Affiliations:** 1 Indiana University Division of Biostatistics, Indianapolis, Indiana, United States of America; 2 Department of Mathematics, Vassar College, Poughkeepsie, New York, United States of America; 3 Department of Biostatistics, Johns Hopkins University, Baltimore, Maryland, United States of America; 4 Indiana University Division of General Internal Medicine and Geriatrics, Indianapolis, Indiana, United States of America; 5 Indiana University Division of Infectious Diseases, Indianapolis, Indiana, United States of America; 6 Academic Model for the Prevention and Treatment of HIV/AIDS (AMPATH), Eldoret, Kenya; 7 Department of Epidemiology and Biostatistics, University of California San Francisco, San Francisco, California, United States of America; 8 National Institute of Allergies and Infectious Diseases (NIAID), Bethesda, Maryland, United States of America; 9 Moi University, School of Medicine, Eldoret, Kenya; Instituto de Pesquisa Clinica Evandro Chagas, FIOCRUZ, Brazil

## Abstract

**Background:**

Monitoring and evaluation (M&E) of HIV care and treatment programs is impacted by losses to follow-up (LTFU) in the patient population. The severity of this effect is undeniable but its extent unknown. Tracing all lost patients addresses this but census methods are not feasible in programs involving rapid scale-up of HIV treatment in the developing world. Sampling-based approaches and statistical adjustment are the only scaleable methods permitting accurate estimation of M&E indices.

**Methodology/Principal Findings:**

In a large antiretroviral therapy (ART) program in western Kenya, we assessed the impact of LTFU on estimating patient mortality among 8,977 adult clients of whom, 3,624 were LTFU. Overall, dropouts were more likely male (36.8% versus 33.7%; p = 0.003), and younger than non-dropouts (35.3 versus 35.7 years old; p = 0.020), with lower median CD4 count at enrollment (160 versus 189 cells/ml; p<0.001) and WHO stage 3–4 disease (47.5% versus 41.1%; p<0.001). Urban clinic clients were 75.0% of non-dropouts but 70.3% of dropouts (p<0.001). Of the 3,624 dropouts, 1,143 were sought and 621 had their vital status ascertained. Statistical techniques were used to adjust mortality estimates based on information obtained from located LTFU patients. Observed mortality estimates one year after enrollment were 1.7% (95% CI 1.3%–2.0%), revised to 2.8% (2.3%–3.1%) when deaths discovered through outreach were added and adjusted to 9.2% (7.8%–10.6%) and 9.9% (8.4%–11.5%) through statistical modeling depending on the method used. The estimates 12 months after ART initiation were 1.7% (1.3%–2.2%), 3.4% (2.9%–4.0%), 10.5% (8.7%–12.3%) and 10.7% (8.9%–12.6%) respectively.

**Conclusions/Significance Abstract:**

Assessment of the impact of LTFU is critical in program M&E as estimated mortality based on passive monitoring may underestimate true mortality by up to 80%. This bias can be ameliorated by tracing a sample of dropouts and statistically adjust the mortality estimates to properly evaluate and guide large HIV care and treatment programs.

## Introduction

In resource-rich settings such as North America and Europe, use of antiretroviral therapy (ART) has vastly improved the prognosis of persons living with HIV/AIDS [1]–[4]. Over the last five years, international response efforts, such as the Global Fund to fight AIDS, Tuberculosis and Malaria, World Health Organization's (WHO) 3-by-5 program (three million patients under treatment by 2005) and the United States President's Emergency Plan for AIDS Relief (PEPFAR) [5]–[7], have made great strides in increasing the number of HIV infected individuals in resource-poor settings who have access to antiretroviral therapy. Early data indicate that such efforts are having a dramatic impact on the morbidity and mortality of HIV infected individuals in resource-poor settings [8]–[10]. A report from the Institute of Medicine (IOM) evaluating PEPFAR stressed the importance of impact measures and the need to strengthen national monitoring and evaluation systems for health programs [11].

Essential to the identification of the most effective antiretroviral treatment (ART) delivery and cost-effective HIV management strategies for resource-constrained settings is appropriate and efficient monitoring and evaluation of ART care and treatment programs. However, accurate estimates of patient survival and other clinical outcomes have been difficult to obtain, as they are significantly impacted by patient loss to follow-up [12], [13]. These unexplained losses may rise above 40% by twelve months in some cases (see citation [14] and the references therein). In addition to presenting serious clinical and operational challenges, these statistics pose urgent questions about the validity of reported mortality estimates and, by extension, the assessment of the effectiveness of the underlying programs.

In the past, several approaches have been used to ascertain the vital status of patients who have not returned to clinic. The basic level of information gathering, and most common method utilized in resource-poor settings, is a passive surveillance system which relies on family and friends to report patient deaths to clinic personnel. To obtain more comprehensive information, some form of active patient surveillance has been used. This includes telephone contact with the patient or close relatives and acquaintances, home visits, reviews of obituaries, vital statistics registries (where these are available), or a combination of the above [8], [15]. In addition, methods of statistical modeling have been developed to overcome residual biases in the vital status data even in the presence of patient tracing and vital status ascertainment strategies [16].

In two seminal reports, one from the Antiretroviral Treatment in Lower Income Countries (ART-LINC) Collaboration[17] and one from two studies funded by the Agence Nationale de Recherches sur le SIDA (ANRS protocols 059 and 1203) in côte d' Ivoire, reported widely varying rates of loss to follow-up and resulting mortality estimates, depending on whether clinical programs used active or passive patient follow-up systems [8], [15]. Their work strongly argues for the inclusion of active follow-up of patients in HIV clinical care programs to increase clinical surveillance and improved antiretroviral adherence as well as to reduce ascertainment bias in mortality estimates. However, with rapid scale-up of antiretroviral treatment programs and the resultant burgeoning patient population under care and treatment, census patient tracing approaches are virtually impossible for the majority of HIV care and treatment programs in resource constrained settings due to the cost, lack of trained personnel, and other organizational constraints.

By comparison, attempting to trace only a statistical (random or non-random) sample of the lost patients is feasible regardless of the size of the patient population and is thus a scalable alternative to census patient tracing approaches. Combined with statistical adjustment of the information gathered, this approach will generate much improved estimates compared to passive patient follow-up.

## Methods

### Objectives

The primary objective of this paper is to describe how the use of statistical sampling techniques, coupled with medical record infrastructure and a patient tracing program, can be used to a) enable more accurate estimation of mortality particularly in large HIV treatment programs, and b) identify subsets of patients who are at higher risk of being lost to follow-up (LTFU) and who may benefit more from active patient tracing in order to improve their clinical care.

### Participants

#### The Academic Model for the Prevention and Treatment of HIV/AIDS (AMPATH)

A partnership initially established between the Moi University School of Medicine in Eldoret, Kenya Indiana University School of Medicine and Brown Medical School in 2001 [18]. AMPATH is a member of ART-LINC and provided the majority of the data from the East Africa region in the report by Braitstein, Brinkhof and colleagues [8]. AMPATH now provides HIV care and treatment to over 50,000 adults and children living with HIV/AIDS in 19 clinics throughout western Kenya. Patients are managed according to National Kenyan protocols, which are consistent with WHO guidelines. The majority of patients receive free HIV care including basic laboratory services and antiretroviral medicines (ARV). Clinic visits occur monthly for all patients on ARV unless alternative arrangements have been made with their health care provider. Patients who are not yet eligible for treatment are seen monthly or bi-monthly depending on their immunologic status and other factors in their health profile. Standard paper data collection forms are used at enrolment to the program and at each subsequent visit. Data from these forms are entered into the AMPATH electronic Medical Record System (AMRS) [19] by data entry technicians.

Included in this analysis are HIV-positive patients aged 18 years and over, who were enrolled between January 1, 2005 and January 31, 2007 at either of two clinics within the AMPATH system where an active program for tracing patients who had missed clinic visits was initiated. Patients were included regardless of whether they were ART-naïve or they had initiated combination ART (CART).

### Research procedures

#### The AMPATH Outreach Program

Active outreach to patients who miss scheduled appointments started in January 2005 at two of the AMPATH Clinics. These were the clinics at Moi Teaching and Referral Hospital (MTRH), an urban referral hospital located in Eldoret, Kenya's fifth largest city, and Mosoriot, a rural health center which serves a catchment area of approximately 6,000 located about 30 Km from Eldoret. This program has now been extended to the entire AMPATH system.

Outreach workers fill out a locator card for all patients enrolling in the clinical care program. The locator card includes contact information and a map to the patient's residence and is used to find the patient in the event of a missed appointment. The AMRS produces a daily list of patients scheduled for appointments and patients that miss their appointment are listed for outreach based on a three-tier triage algorithm. Adult patients on CART for less than three months are given priority. Outreach efforts for these patients are to commence within 24 hours of a missed appointment with a goal of locating the patient within seven days. For patients receiving CART for over three months, outreach is activated within seven days after a missed appointment. Individuals who do not receive CART are allowed a grace period of 28 days from the missed appointment prior to initiation of outreach activities. At the time of this study, the outreach program maintained a standalone MS Access database that contained data pertaining to every outreach encounter including vital status of located patients and date of death for patients found to be deceased. This database has since become part of the AMRS.

#### Definition of patient dropout

In this study, a patient is considered to be a dropout if the patient has been declared as lost to follow-up by the program. This happens when a patient receiving CART has not kept a visit for more than three months or a patient that is not receiving CART has not come to clinic for more than six months. In addition, any patient for whom outreach was initiated, regardless of whether they had been declared as lost to follow up, is considered a dropout for the present analysis. This includes all patients that have missed appointments that AMPATH attempted to contact. We have not distinguished between patients declared as lost to follow-up and patients that missed appointments and were outreached. This is because the loss-to-follow-up status is not known among patients that have been outreached after a missed appointment but before they have been declared as lost to follow-up (i.e., earlier than three months for patients on CART or before six months for patients not receiving CART). For this reason, dropout rates reported here are much higher than the loss-to-follow-up rates previously reported for the AMPATH program [10].

### Ethics

All patients in the study provided locator information as described above. This is part of the standard of care at AMPATH. Use of these data, which were routinely collected as part of the AMPATH care protocol, was approved by both the Indiana University Institutional Review Board (IRB) and the Moi University Institutional Research and Ethics Committee (IREC).

### Statistical methods

Descriptive statistics were produced. Patient subgroup sizes (e.g., gender) were compared between dropouts and non-dropouts by chi-square tests. Continuous measures such as CD4 count were compared via the Kruskal-Wallis test. Estimates of time until an event (e.g., time from enrollment until CART initiation) were produced by the method of Kaplan and Meier, with time zero (baseline) being defined as the date of enrollment in the AMPATH program. Comparisons of these times between dropouts and non-dropouts were performed by the log-rank test. A Cox proportional-hazards model was used to assess the impact on time to loss-to-follow-up of a number of measures obtained from the patients prior to dropping out. These are WHO stage, CD4 count at enrollment, gender, age and type of clinic attended (urban versus rural). In addition, we have stratified the analysis to account for CART start status (i.e., CART≤3 months or CART>3 months) because this factor summarizes a number of patient and disease-related issues that may not be captured by the other factors considered above. These analyses were implemented with SAS version 9.1 (SAS Institute, Cary, NC).

Statistical estimation of mortality follows methods described by Frangakis & Rubin [20], based on the concept of “double sampling [21]. Briefly, their method selects a random sample of dropouts, on which active follow-up is performed and their vital status is determined. Survival estimates are then produced as an average of the hazard of death between dropouts and non-dropouts weighted for the relative size of the dropout and non-dropout group. This double sampling approach attempts to overcome the bias that is generated when analysis is based only on observed data (such as when only considering the vital status information passively recorded). This bias occurs because patients that are maintained under care are not representative of the group that is lost to follow-up. More importantly, the authors show that the differences in survival in these two groups cannot be accounted for by adjusting for measurements obtained prior to dropping out. Simple pooling of the deaths that are discovered through routine follow-up and those detected by active patient tracing is not sufficient and the resulting estimates from such a naïve approach may still seriously underestimate overall mortality. By contrast, the Frangakis & Rubin double-sampling method also corrects the estimates that are produced when double sampling (i.e., outreach) data are incorporated into the estimates. We implemented the Frangakis & Rubin methodology by using software specifically written for this analysis. This software can be provided by Drs. Frangakis and An upon request.

#### Survival time

For patients that were deceased, we calculated survival time as the time from enrollment to the date of death. For deceased patients with missing death dates, we imputed a death date from other patients with a known death date and matching CD4 counts at enrollment. If no matches were found and the patient had a baseline WHO stage, then we searched among the deceased patients with available death dates and matching WHO stage. We then imputed a survival time from among this subset. If the above step found no matches, then we matched based on available death information from patients that started CART at a similar time from enrollment.

#### Censoring time

For patients who dropped out, were double-sampled, were alive and returned to clinic, we defined “censoring time” to be the time from enrollment to the date of the most recent visit after outreach. For those found alive but did not return to clinic we imputed time from last visit (before dropout) to last visit after outreach from observed data, matching in a similar manner to that described for the death date above. We then added this imputed time to the observed time from enrollment to last visit to obtain an imputed date of last visit after outreach. Otherwise, “censoring time” was the time from enrollment to the end of the study (1/31/07), the usual administrative censoring time.

#### Baseline CD4 and WHO stage

A CD4 count and WHO stage measurement were considered to be a “baseline” measurement if the measurement were taken within 3 months after enrollment. If there were multiple measurements during this period, the one closest to enrollment was selected as “baseline”. Missing CD4 count and/or WHO stage were imputed according to the following:

- If CD4 count but not WHO stage were missing, CD4 count was imputed from among patients with similar WHO stage and matched CART start status (i.e., CART≤3 months or CART>3 months).
- If WHO stage but not CD4 count were missing, WHO stage was imputed from among those with similar CD4 (stratified as <200 or ≥200 cells/ml) and matched CART start status.
- If both CD4 and WHO stage were missing, imputation of WHO stage and CD4 count was obtained from among patients with matching CART start status.

#### Methods of mortality estimation

We compared four methods for estimating mortality. The first method is the usual passive follow-up Kaplan-Meier estimate that is based on observed deaths only (Method 1). The mortality estimates produced by Methods 1 would typically be those reported from passive follow-up programs. In addition, a Kaplan-Meier estimate based on all deaths (both those recorded passively as well as those discovered through patient tracing) was produced (Method 2). In this method all vital status information is pooled without consideration whether it was obtained by passive or active follow-up efforts. These results would be routinely reported by active-follow-up programs with no statistical adjustment. We also produced a mortality estimate as the weighted average of the Kaplan-Meier survival estimates between dropout and non-dropout groups (Method 3). This method should be an improvement compared to Methods 1 and 2 because it assigns the proper weighting (invariable higher weight) to deaths discovered through patient outreach. This method however has been shown by Frangakis and Rubin to be biased [20]. The fourth method is the one proposed by Frangakis and Rubin (Method 4). This method produces a survival estimate as a function of the weighted average of the hazard of death between dropouts and non-dropouts instead of a weighted average of the survival. This method also weighs deaths that are observed from outreach usually by a higher weight than passively observed deaths. The authors showed that this method produces unbiased estimates of patient survival.

## Results

### Baseline characteristics

There were 8,977 adult patients enrolled in the two participating sites between January 1, 2005 and January 31, 2007. Of these, 3,624 (40%) had missed clinic visits over this period, including 64% on CART and 36% not on CART. Outreach efforts were initiated for 1,143 (31.5%) of these and 621 (54.3%, 17.1% overall) were successfully located. Reasons why patients were not contacted through the Outreach Program include insufficient or inaccurate locator information or the patient returned to clinic prior to the initiation of outreach procedures. Unfortunately, the reason for not initiating outreach was not included in the outreach database at the time. It has been added to the newer version of the database.

Patient characteristics at enrollment into AMPATH are shown in Table 1. Imputed data generated for the Frangakis & Rubin analysis are not reflected in the patient characteristics data shown in the table. Approximately 35% of the patients were male, 73% of all patients attended Moi Teaching and Referral Hospital (the urban clinic) and 43.6% were WHO stage 3 or 4 at enrollment. The overall median age was 35.5 years (IQR = 29.6–42.3). The overall median CD4 count (available data only) at baseline was 183 cells/ml (IQR 75–352) and the median time from enrollment to CART initiation was 9.9 weeks (95% CI 9.6–10.3 weeks). Eventual dropouts were slightly more likely to be male (36.8% versus 33.7%; p = 0.003), and younger than non-dropouts (35.2 versus 35.8 years old; p = 0.020). They were also sicker at baseline compared to non-dropouts as determined by significantly lower median CD4 count at enrollment (162 versus 201 cells/ml respectively; p<0.001) and higher WHO stage (percent 3–4 stage 47.5% versus 41.1%; p<0.001). As a result, eventual dropouts were initiated on CART sooner after enrollment than non-dropouts (median time to CART 6.9 versus 12.1 weeks; p<0.001). A smaller percent of patients attending the urban clinic ultimately dropped out during the study compared to patients attending the rural clinic (70.3% versus 75.0% respectively; p<0.001).

**Table 1:** Patient characteristics and comparison between dropouts and non-dropouts.

Characteristic		Patient subgroup		p-value
	Total	Dropouts	Non-dropouts	
	N = 8,977	N = 3,624	N = 5,353	
Male Gender				0.003
N (%)	3,138 (35.0%)	1,334 (36.8%)	1,804 (33.7%)	
Urban clinic				<0.001
N (%)	6,561 (73.1%)	2,547 (70.3%)	4,014 (75.0%)	
Baseline WHO stage 3–4				<0.001
N (%)	3,010* (43.6%)	913 (53.0%)	2,097 (40.5%)	
Baseline CD4 count (cells/ml)				<0.001
Median (IQR)	183 (75–352)	162 (56–325)	201 (86–367)	
Age (years)				0.020
Median (IQR)	35.5 (29.6–42.3)	35.2 (29.2–42.1)	35.8 (29.9–42.4)	
Time until CART start (weeks)				<0.001
Median (95% CI)	9.9 (9.6–10.3)	6.9 (6.1–7.7)	12.1 (11.7–13.4)	

Frequencies were compared via Pearson's chi-square test. Continuous factors were compared via the Kruskal-Wallis test. Median times from enrollment until CART start were estimated via the method of Kaplan and Meier and were compared by the log-rank test. IQR = Inter-quartile range.

* Out of 6,900 total patients (5,178 non-dropouts and 1,722 dropouts) with WHO stage recorded within three months of enrollment.

### Impact of baseline factors on dropout risk and on being successfully located

Table 2 lists the results of the Cox proportional hazards model. The impact of a number of baseline factors on dropout was considered based on their statistical significance in univariate analyses. The factors that were included in the model were: male gender (hazard ratio [HR] = 1.065; p = 0.005), advanced WHO stage (stage 3 or 4 versus 1 or 2; HR = 1.101; p<0.001) and CD4 count (50–200 and >200 versus <50 cells/ml; HR = 0.935; p = 0.027 and 0.918; p = 0.007 respectively). All remained independently associated with higher risk of dropout. By contrast, CART initiation at enrollment and attendance to an urban versus a rural clinic were not significantly associated with the chance for dropout.

**Table 2:** Results of the Cox proportional hazards model of patient dropout.

Factor	Hazard ratio (95% CI)	P value*
Gender (male versus female)	1.065 (1.021–1.109)	0.005
WHO stage (3/4 versus 1/2)	1.101 (1.057–1.145)	<0.001
CD4 count (cells/ml)		
CD4≥200 cells/ml	0.918 (0.856–0.980)	0.007
50≤CD4<200 cells/ml	0.935 (0.875–0.995)	0.027
CD4<50 cells/ml	1.00	
ARV at first visit	0.972 (0.897–1.047)	0.970
Urban clinic	0.999 (0.924–1.076)	0.460

* Wald test.

We generated a dropout risk score based on the Cox proportional hazards model presented in Table 2 and compared this risk score between various dropout subgroups. The dropouts for whom outreach was not attempted had a slightly higher risk score for dropping out compared to those dropouts that were outreached (p = 0.032). By contrast, among the latter group, the successfully located dropouts had somewhat higher risk score for dropping out compared to the outreached dropouts that were not successfully located (p = 0.036).

### Estimates of patient mortality

**Figure 1:**
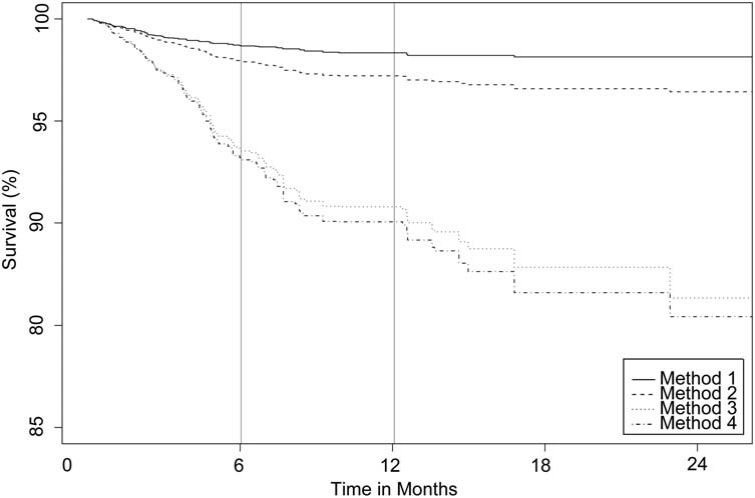
Kaplan-Meier estimates of patient survival based on methods described in Table 3. Time is in months since enrollment.

**Figure 2:**
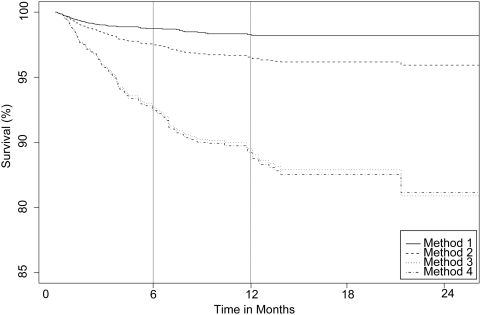
Kaplan-Meier estimates of patient survival based on methods described in Table 3. Time is in months since start of CART.

We present two analyses based on two definitions of “baseline” as the date of enrollment and the date of ART initiation. Results from the four methods of estimating patient mortality are shown in Table 3 and summarized in Figures 1 and 2. With the exception of Method 1 that involves 126 deaths discovered through routine follow-up procedures, the remaining three methods consider all 230 deaths that were discovered through both patient tracing and passive follow-up. The overall mortality 12 months after enrollment produced by Method 1 is 1.7% (95% CI 1.3%–2.0%). The overall one-year mortality generated by Method 2, which pools all deaths regardless of whether they were discovered through active or passive follow-up, is 2.8% (2.3%–3.1%). The one-year mortality estimate based on all deaths but stratifying according to the relative size of the dropout and non-dropout groups (Method 3) is 9.2% (7.8%–10.6%). Method 4, which statistically adjusts the estimate of one-year mortality by the Frangakis & Rubin method [20] is 9.9% (8.4%–11.5%). Mortality at one-year post ART initiation estimated by Method 1 was 1.7% (1.3%–2.2%), revised to 3.4% (2.9%–4.0%) by Method 2, 10.5% (8.7%–12.3%) by Method 3 and 10.7% (8.9%–12.6%) by Method 4.

**Table 3:** Mortality estimates (%, 95% CI) at one year from enrollment and one year from ART initiation. Overall estimates based on entire cohort.

Method	Mortality since enrollment (%)	Mortality since ART start (%)
**Passive follow-up**		
1. Naïve estimate (no statistical adjustment)	1.7 (1.3–2.0)	1.7 (1.3–2.2)
**Combined passive and active follow-up**		
2. Naïve estimate (no statistical adjustment)	2.8 (2.3–3.1)	3.4 (2.9–4.0)
3. Stratifying on dropout group		
a. Kaplan Meier estimate (dropouts)	20.0 (2.6–23.5)	20.4 (2.9–26.7)
b. Kaplan-Meier estimate (non-dropouts)	2.2 (1.8–16.6)	2.3 (1.7–18.0)
c. Weighted Kaplan Meier estimate*	9.2 (7.8–10.6)	10.5 (8.7–12.3)
4. Based on Frangakis & Rubin method	9.9 (8.4–11.5)	10.7 (8.9–12.6)

* Combined estimates were produced as a weighted average of the individual (dropout versus non-dropout) estimates, taking into account the relative size of the two groups.

## Discussion

We present data from AMPATH, a large PEPFAR-funded HIV clinical care program in western Kenya. AMPATH has implemented an active follow-up program that attempts to locate as many patients as possible among those that have discontinued care. Analysis of these data allows adjustment of mortality estimates based on information obtained on patient dropouts that were located as part of the AMPATH outreach program.

Comparison between mortality estimates reveals some interesting results. A significant number of patients miss at least one clinical visit (40% over almost two years in our cohort). The majority of these will be lost to follow-up. Since risk factors for patient dropout (lower CD4 count or higher WHO stage at enrollment) are also risk factors for mortality, estimates that do not account for patients lost to follow-up are bound to severely underestimate the true extent of mortality and conversely overestimate the positive impact of a care and treatment program. This is not a novel observation. The significance of this finding is the size of the differences in mortality estimates produced by statistically adjusted methods. The mortality estimate produced by relying on passive follow-up (Method 1) was 1.7% one year after enrollment in the program compared to 2.8% when all known deaths are incorporated into the analysis (Method 2). This almost two-fold difference strongly supports inclusion of active follow-up for at least a sample of the patient dropouts. This has been consistently reported in similar settings [8], [15], [22]. Review of the results of Methods 3 and 4 however, suggest that incorporating active follow-up to the monitoring and evaluation of HIV care and treatment programs is only a partial solution to the problem. The corresponding one-year mortality estimates produced when the relative size of the dropout and non-dropout groups is taken into account (Methods 3 and 4) are much higher than the estimates produced by naïvely pooling the information without regard to its source (passive versus active follow-up). The one-year mortality estimate produced by Method 3, which stratifies according to the relative sizes of the dropout and non-dropout groups, was 9.2% and the one produced by Method 4 was 9.9%. These are much higher than 2.7% despite the fact that all three estimation methods incorporated the same death information.

A further example in which unadjusted data can skew program estimates of mortality concerns AMPATH's contribution to a recent ART-LINC collaboration [23], where (see entry under “Eldoret” in Table 1 in their paper) the six-month mortality after start of ART is 0.7%. These data include some deaths ascertained through active follow-up so they correspond to mortality estimates somewhere between Method 1 and 2 in our paper. The mortality rate estimated through Methods 3 and 4 in this study (extrapolated from the Kaplan-Meier curves of Figure 2) is 7%. Thus, the magnitude of bias that unadjusted data such as these can introduce to death reporting can be significant.

Estimated mortality according to Methods 1 and 2 is significantly different from Methods 3 and 4. These differences do not abate with time but instead become increasingly pronounced as shown in Figures 1 and 2. The reason for this divergence with time is that a higher proportion of late deaths is established through outreach and is thus up-weighted by the statistical methods. This accelerates mortality increases in the statistically adjusted survival curves. The reason for the differences in one-year mortality estimates between Methods 1 and 2 can be further elucidated by inspection of the results in Table 2. Patients that ultimately dropped out were more frequently men with more advanced disease at enrollment as determined by their higher WHO stage. This implies that the subset of patients that were not lost to follow-up is not representative of the entire patient population. Thus, any estimates generated by patients under observation will be biased. The disparity between Method 2 and Methods 3 and 4 is due to the fact that while 126 deaths were observed among 5,363 non-dropouts, 124 deaths were observed among the 621 patient dropouts that were successfully located by outreach. Thus, mortality among dropouts is much higher and consequently simple pooling of the information results in a significant underestimate of the overall mortality. This observation indicates that the considerable investment of incorporating active follow-up to a care and treatment program will have very limited value for estimating mortality unless monitoring and evaluation of resulting data is accompanied by appropriate statistical adjustment.

The impression from mortality estimation adjustments one year after ART initiation is similar. The naïve estimate produced by Method 1 is 1.7%, revised to 3.4% when all deaths are pooled (Method 2), and revised further to 10.5% and 10.7% by using statistical adjustment (Methods 3 and 4 respectively). The mortality one year after ART is slightly higher than the estimate obtained one year after enrollment because a sizable proportion of the patient population did not require CART at entry because of less advanced disease and thus had lower risk of death during that period. By contrast, patients requiring ART had more advanced disease (a prerequisite for being eligible for treatment) and, at least in the short term, higher risk of death.

To further understand the impact of dropout on estimates of mortality, we refer to ANRS protocols 059 and 1203, both initiated prior to the wide availability of CART [15]. In that study, the investigators implemented progressively more intensive patient outreach methods to locate as many of the lost patients as possible. Each successive round of patient tracing located a larger proportion of patients and resulted in progressively higher one-year mortality estimates. The estimated one-year mortality rate increased from 10.5%, based on passive follow-up when only 59% of dropouts were accounted for, to 12.9% when 64% of dropouts were accounted for. With the most intensive follow-up procedures, 81% of dropouts were accounted for, resulting in a one-year mortality estimate of 19.6%. Their analyses did not include any statistical adjustment for the dropouts that were not accounted for even through the most intensive outreach efforts, so the suggestion is that, had the deaths discovered through outreach been weighed appropriately, the true one-year mortality rate might have been higher still. The impression from this report is that even active follow-up programs that successfully locate a large proportion of their dropout patients may produce inaccurate estimates of the true mortality of their cohort unless some statistical adjustment is used.

Our results have both similarities with as well as important deviations from published reports in similar settings. The results from Method 1 are similar to the mortality estimate of 2.3% one year after CART initiation in a “passive follow-up cohort” reported by Braitstein, Brinkhof and colleagues from the ART-LINC collaboration [8]. The 95% confidence interval from that report (1.5%–3.2%) covers the estimate of 1.7% mortality at one year after CART initiation produced by Method 1. The one-year mortality estimate of 3.4% produced by simple pooling of dropout and non-dropout death information (Method 2) is lower than their one-year mortality estimate of 6.4% generated from active follow-up programs. The reason for this may be that the active follow-up programs in ART-LINC successfully located a much higher proportion of dropouts than the one implemented by AMPATH (which also provided data for that report as part of the ART-LINC collaboration). As previously reported, patients that are harder to find may have higher risk of death [15]. Thus, it is expected that the pooled estimator of mortality that was derived from the smaller proportion of patient dropouts that were outreached and located by AMPATH, likely seriously underestimates true mortality. When adjusting for the relative size of the dropout and non-dropout subgroups, the resulting mortality estimates one year after enrollment (9.2% and 9.9% produced by Methods 3 and 4 respectively) and one year after CART initiation (10.5% and 10.7%), are significantly higher than the one-year mortality estimates obtained from the ART-LINC active follow-up programs. The reason for this likely is that not every patient lost from observation was accounted for by the ART-LINC active follow-up programs (personal consultation with Drs. Braitstein and Brinkhof). Thus, simple pooling of death data, even from programs such as ART-LINC, that follow-up a large proportion of their dropout patients, may still produce biased results. Since the publication of their article, the ART-LINC collaborators have updated and re-analyzed their data by relying on programs with more successful active follow-up and have produced higher one-year mortality estimates more in line with our statistically adjusted ones [24].

### Limitations

There are several limitations in our study. The most important one is that the Frangakis & Rubin method we used to produce the adjustment of one-year mortality estimates assumes that a random sample was selected among the dropout patients and everyone from that sample was located. By contrast, AMPATH attempted to locate 1,143 patients out of the 3,624 dropouts (31.5%) of whom 621 were successfully located (54.3% of the outreached patients or 17.1% overall). We have made the tacit assumption that the group of patients that was outreached and the subgroup that was successfully located were both representative of the entire dropout population. The ANRS 059/1203 study results [15] suggest that harder to locate AMPATH patients may have been at higher risk of death and that difficulties in locating some patients may reflect latent subpopulations among the dropouts. It is thus possible that our mortality estimates may still underestimate the true mortality rate since the dropouts that were not accounted for may have higher risk of death than the dropouts that were located. As reported in the Results Section, the dropouts that were not outreached had a slightly higher risk score for dropping out compared to those dropouts that were outreached. Since the factors entering in the development of that score are also factors associated with poor patient outcome, this difference in risk for dropout may imply that mortality was underestimated by the AMPATH data. By contrast, the successfully located dropouts had somewhat higher risk score for dropping out compared to the outreached dropouts that were not successfully located. This observation implies that the mortality estimates based on patients under observation and successfully located dropouts might have overestimated mortality. Given these observations, we expect that the statistical estimates of mortality presented earlier, represent a largely accurate reflection of the true mortality rate.

Our findings lead to the inescapable conclusion that both active follow-up and appropriate statistical modeling must be employed in combination to account for measurable and non-measurable factors affecting mortality. With rapid scale-up of HIV care and treatment programs, census approaches that canvass all lost patients (such as those reported by Anglaret and colleagues [15] and Bisson et al. [22], from cohorts of 545 and 410 patients respectively) are inconceivable for large cohorts following tens of thousands of patients. Even in the rare cases where this is attempted, the resulting estimates would be biased since invariably a large portion of patients lost to follow-up will not be located. Thus, tracing a random sample of the dropouts and adjusting the resulting estimates statistically is the only viable way to monitor and evaluate increasingly large antiretroviral programs as rapid treatment scale-up continues in Africa and the developing world.
